# Patterns of variations in dorsal colouration of the Italian wall lizard *Podarcis siculus*

**DOI:** 10.1242/bio.058793

**Published:** 2021-10-06

**Authors:** Federico Storniolo, Marco A. L. Zuffi, Alan J. Coladonato, Loris Di Vozzo, Gianni Giglio, Andrea E. Gini, Francesco L. Leonetti, Simone Luccini, Marco Mangiacotti, Stefano Scali, Federico Abate, Emilio Sperone, Irene Tatini, Roberto Sacchi

**Affiliations:** 1 Museo di Storia Naturale, Università di Pisa, Via Roma 79, Calci (PI) 56011, Italy; 2 Dipartimento di Scienze della Terra e dell'Ambiente, Università di Pavia, Viale Tamarelli 24, Pavia I-27100, Italy; 3 Dipartimento di Biologia, Ecologia e Scienze della Terra, Università della Calabria, Via Pietro Bucci, Arcavacata di Rende, Cosenza 87036, Italy; 4 Faculty of Sciences, Scuola Normale Superiore, Piazza dei Cavalieri 7, Pisa 5616, Italy; 5 Museo di Storia Naturale, Corso Venezia 55, Milano 20121, Italy

**Keywords:** *Podarcis siculus*, Colour pattern, Sexual signalling, Seasonality, Sexual dichromatism, HSV colour system

## Abstract

Research on animal colouration has grown exponentially in the last decade thanks to multidisciplinary approaches. Most studies are focused on trade-offs between communication and mimicry, which represent the two main constraints and drivers of the evolution of body colourations. Reptiles are excellent model species for investigating this field of study and lizards in particular show great variability of body colourations and their functions. We studied the lizard *Podarcis siculus*, analysing the variations of dorsal colour of three populations and obtained clear patterns of seasonal and ontogenetical variation of dorsal colour. According to baseline colour, males were greener and brighter than females, although no difference in saturation was recorded. According to seasonal variations, analyses showed that both sexes significantly vary in colour over the year: males reached higher peaks of hue and saturation later than females during spring, while females showed higher peaks of brightness and reached earlier similarly to hue and saturation. Ontogenetic variations were recorded only in males, which become greener, less bright and saturated with growing size. Therefore, our results suggest the occurrence of two opposing strategies in colour expression between sexes: males’ dorsal colouration plays a major role in communication, while females are more crypsis-oriented.

## Abstract (precis)

**Summary:** This research paper focuses on the dorsal chromatic variations in Mediterranean lizards, analysing the effect of seasonality and ontogeny.

## INTRODUCTION

Research on animal colouration has been carried out intensively in the past few decades and nowadays it is a fast-growing interdisciplinary branch of zoology that involves a wide variety of biological sectors, namely genetics (Fulgione et al., 2004; Brenig et al., 2013), developmental biology (Wilson et al., 2007), reproductive biology (Wellenreuther et al., 2014) and feeding ecology (Schaefer et al., 2008), as well as all of their reciprocal interconnections. As a matter of fact, the evolution of body colouration is more often the result of the interaction between synergic and contrasting pressures rather than the outcome of one single factor (Endler, 1981). Therefore, it has been approached under various perspectives with the aim of investigating what drives the evolution and selection of specific body colourations. The task of this field of research is to understand the mechanisms involved in colour expression and perception in order to define the role of body colourations as a means of communication (Cuthill et al., 2017). Physiological studies focused largely on the mechanisms and constraints of colour expression, which is mainly determined by the innate production of pigments or, alternatively, by their acquisition from the environment via the trophic chain (Hill et al., 1994; Shawkey and D'Alba, 2017). Conspicuously coloured animals have to compromise between the communicative efficiency of a manifest colouration and the energetic cost of the production of pigments (Hill and Montgomerie, 1994). Another aspect of colour as a communication tool consists of the receiver's sensory perception that could be involved in a process of close evolutionary correlation between the colour pigments of the emitter and the visual receptors of the receiver (Lind et al., 2017; Renoult et al., 2017). Body colouration is also a highly informative trait for both inter- and intraspecific interactions as it may serve as an honest signal to warn potential threats (aposematism) (Mappes et al., 2005), attract mating partners (female choice based on the potential quality of the male) (Slagsvold and Lifjeld, 1988; LeBas and Marshall, 2000; Bajer et al., 2010) or to discriminate the sex of conspecifics (Cooper and Burns, 1987). Contrastingly, conspicuous colourations tend to increase the chance of being detected by predators causing fitness reduction. Thus, the evolution of cryptic and disruptive colour patterns, alongside deterrent or elusive behaviours, can be the adaptive response to compensate higher predation rate (Forsman, 1995; Forsman and Shine, 1995; Stuart-Fox et al., 2003).

Reptiles show noticeable variability of body colourations and patterns, and hence they are excellent model species in this research perspective. In the common adder (*Vipera berus*) melanism is positively correlated to higher reproductive success thanks to increased thermoregulatory efficiency, notwithstanding its negative effects on survivorship due to a higher chance to be detected by avian predators (Andrén and Nilson, 1981). Some lizards, as reported for *Sceloporus grammicus*, discriminate among individuals belonging to the same or different groups thanks to specific colour polymorphisms. Such variability is often characteristic of ventral parts of the body such as belly and throat, which are typically less visible than dorsal ones, thus compromising with potential predation risks (Bastiaans et al., 2014). Colour polymorphisms also influence social interactions and contests. The red males of the polymorphic Australian painted dragon *Ctenophorus pictus* tend to win most contests against yellow competitors; in keeping with the fact that red is commonly considered a sign of dominance, in this case, red colour has also the effect of reducing the aggressive response of the yellow counterparts (Healey et al., 2007).

The Italian wall lizard *Podarcis siculus* is an endemic lacertid of the Italian peninsula and the Adriatic basin and an efficient invasive species in other European and not European countries (Burke et al., 2002; Ribeiro and Sá-Sousa, 2018; Damas-Moreira et al., 2019). This lizard is dorsally green-brown for most of its activity period (March to October) and males especially show a green to azure gular region at the peak of their reproductive season (Corti et al., 2011). Its spreading success within and outside its original natural distribution range can be explained by its great adaptability in terms of trophic ecology (Zuffi and Giannelli, 2013), thermoregulatory efficiency (Kapsalas et al., 2016), and tolerance to anthropogenic environmental modifications (Mangiacotti et al., 2013). Although body colourations within the *Podarcis* genus have been widely investigated, most studies on Italian species have been carried out on *P. muralis* and its polymorphisms (Capula et al., 2009; Sacchi et al., 2013) while *P. siculus* has been investigated to a lesser extent under this perspective (Fulgione et al., 2008). Nonetheless, seasonal colour variation has been analysed in a recent paper, which showed that dorsal colourations match the background colour of the environment according to seasonal changes in vegetation colour, supporting the hypothesis that dorsal colouration can play a significant role in camouflage (Pellitteri-Rosa et al., 2020). However, dorsal (green) colourations in Lacertids are often involved in intersexual competition (Martín and López, 2010), where the intensity of the colouration correlates with male quality and forecasts the hierarchies of dominance in males (Olsson, 1994; Martín and López, 2009). Furthermore, darker colourations such as melanism are sometimes related to stronger immunity responses and resistance to ectoparasites (Baeckens and Van Damme, 2018). Since the breeding cycle of the Italian wall lizard matches the seasonal chromatic peak of green in local vegetation (males are most active in spring), the hypothesis that the dorsal colouration might play a role in intraspecific communication, although unlikely given that dorsal colourations are mostly camouflage-oriented, cannot be excluded. Notably, in earlier studies, neither sexual differences in dorsal colouration over the season nor the effect of body size on colour intensity have been investigated in detail (Pellitteri-Rosa et al., 2020). Therefore, this paper will focus on seasonal dorsal chromatic variations in three populations of *P. siculus* from northern and southern Italy, and on the possible effect of sex and body size on such variations.

## RESULTS

**Fig. 1.**
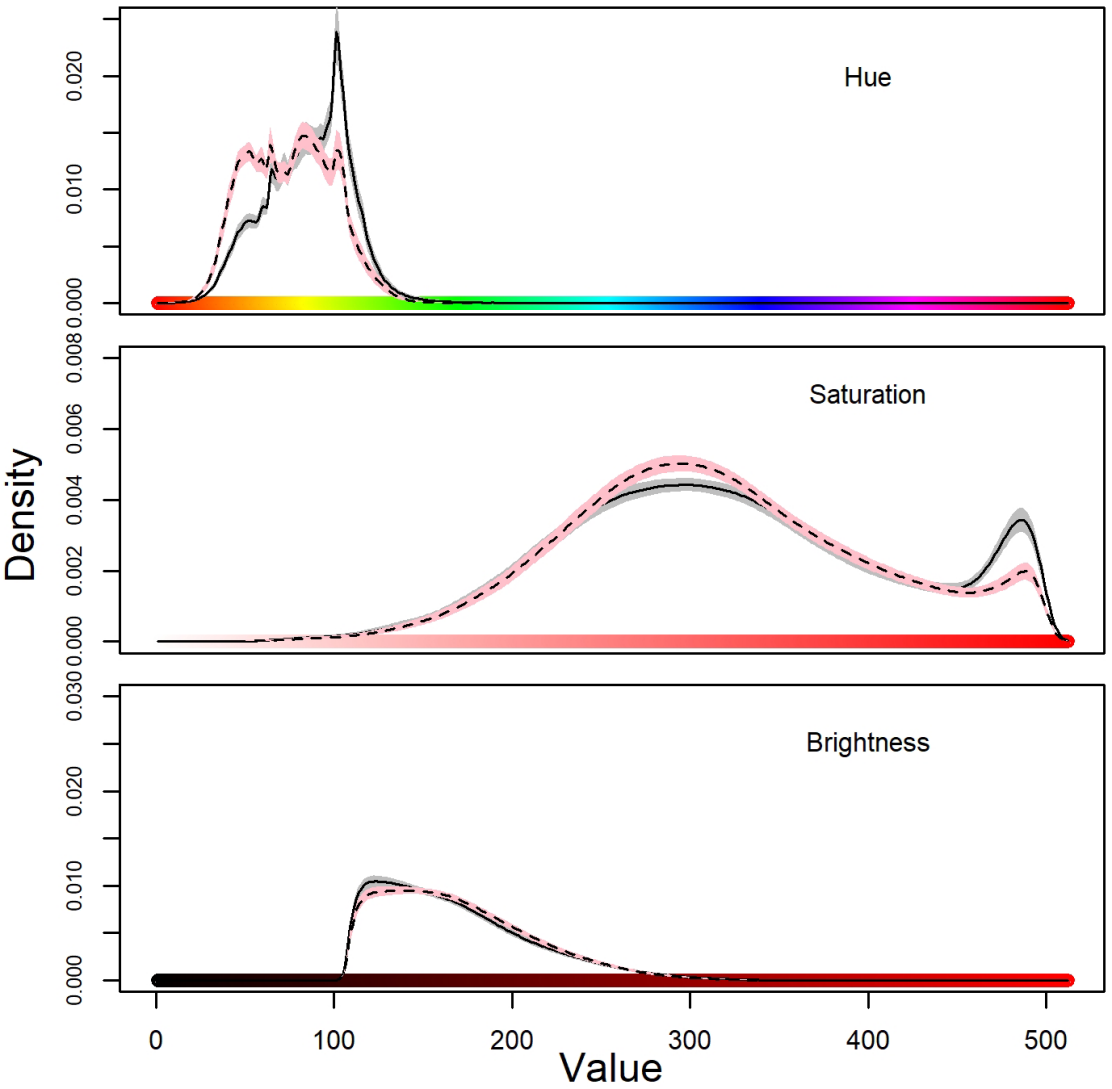
**Distribution frequencies of hue, saturation and brightness of body colouration of Italian wall lizards.** Solid lines and dark grey areas are for males, dashed lines and pink areas are for females. Lines are means and area represent HDI_95_.

The first step of the analysis consisted in calculating the mean values for hue, saturation and value and then computing their frequency distribution from the total number of pixels analysed; as shown in Fig. 1, males (m) tend to be generally greener than females (f), to show a higher amount of pigment, and to be slightly brighter.

**Fig. 2.**
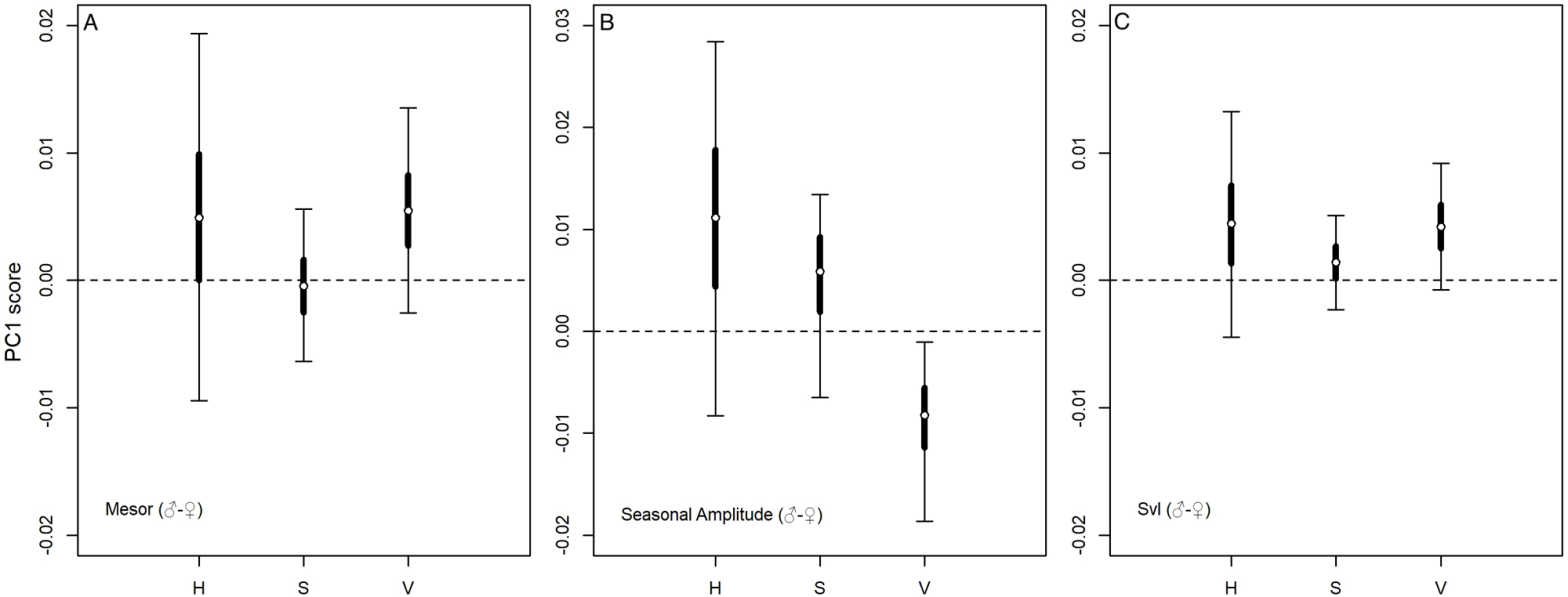
Differential m-f posterior distribution values of the first component score of baseline colour (A), seasonal variation (B) and size (C) for each colour parameter (hue, saturation, value).

The Bayesian mixed models supported the occurrence of a difference in the baseline colour (Mesor) between males and females, as the posterior distributions of the difference between sexes (m-f) deviated from zero for H and V, but not for S values (Fig. 2A). In detail, males were greener (*P*_m>f_=0.75) and more brilliant (*P*_m>f_=0.91) than females, but dorsal colouration was as saturated in males as in females (*P*_m>f_=0.56).

**Fig. 3.**
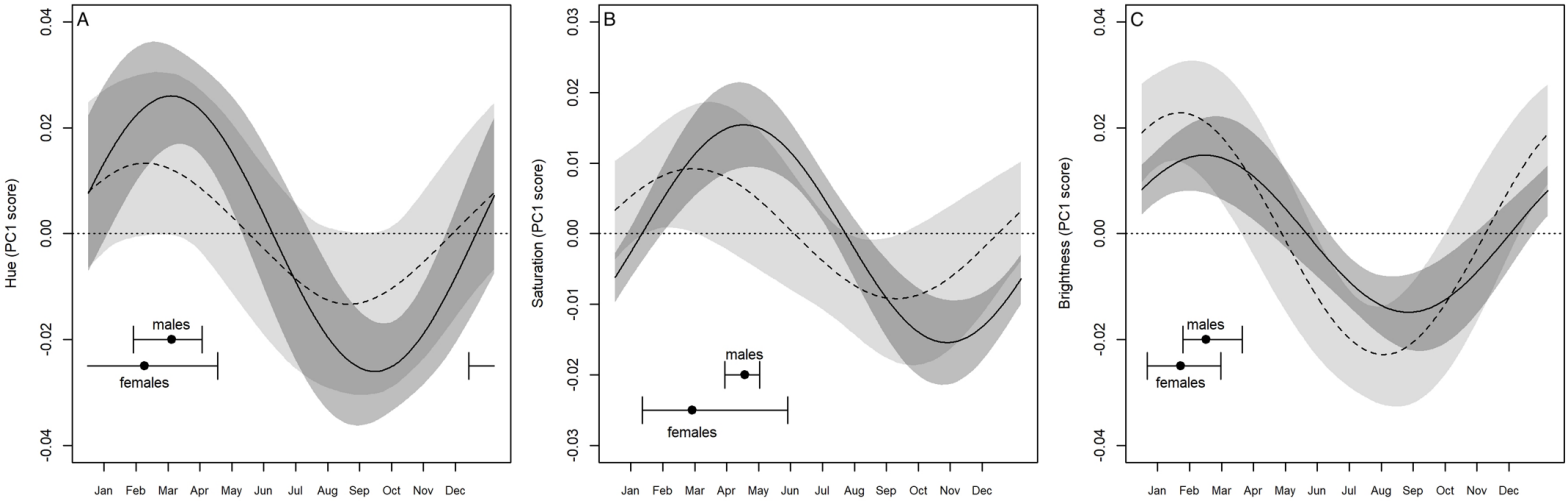
**Effect of seasonality on hue (A), saturation (B) and value (C) differential patterns in males (solid lines) and females (dotted lines).** Grey areas are 95% credibility intervals.

**Fig. 4.**
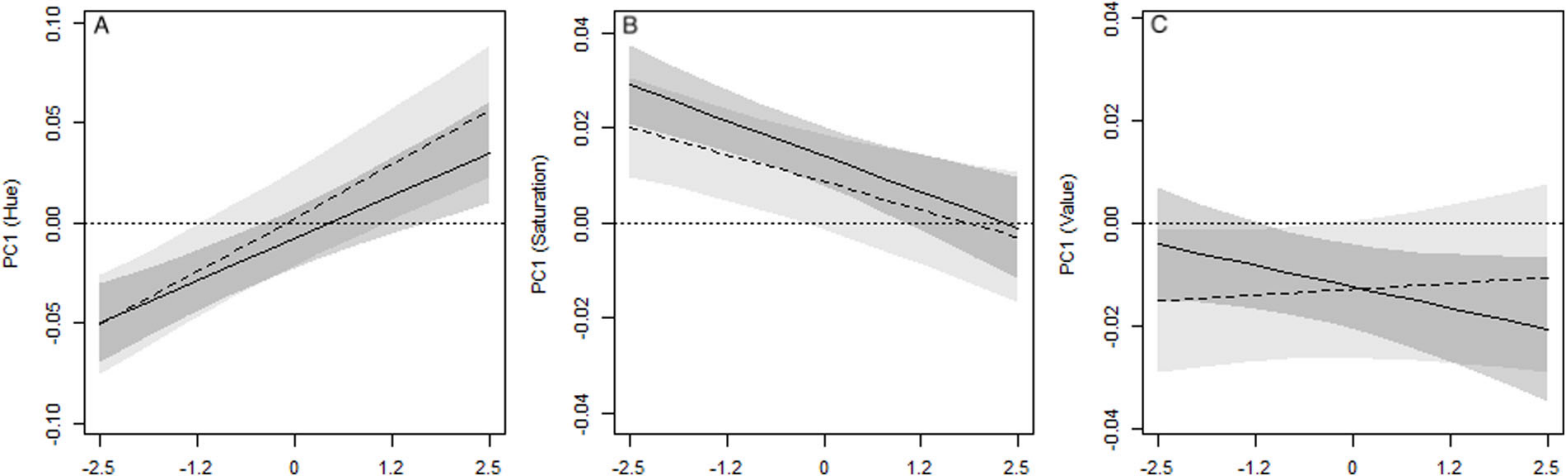
**Effect of size (standardized SVL) on hue (A), saturation (B) and value (C) differential patterns in males (solid lines) and females (dotted lines).** Grey areas are 95% credibility intervals.

The analysis of the Cosinor components on the score of the first component of hue (i.e. the seasonal effect) showed that the amplitude was not null, in both males and females (males: P_A>0_=0.999; females: P_A>0_=0.999, Table 1). This result supported the occurrence of a seasonal pattern of green expression in both sexes. However, males showed a seasonal amplitude larger than females (*P*_m>f_=0.87, Fig. 2B), and reached the peak on 16 March (HDI_95_: 11 February, 12 April, Fig. 3A), whereas females did it on 21 February (HDI_95_: 8 December, 26 April, Fig. 3A). A further relevant effect on greenness was detected for body size, but only in males. Indeed, the hue PC1 score of males increased with body size, but it did not in females (Table 1). Consequently, the posterior probability of the differential effect of size between sexes deviated from zero (*P*_m>f_=0.84, Fig. 2C). This result suggested a positive relation between size (age) and green expression only in males (Fig. 4A).

**Table 1.**
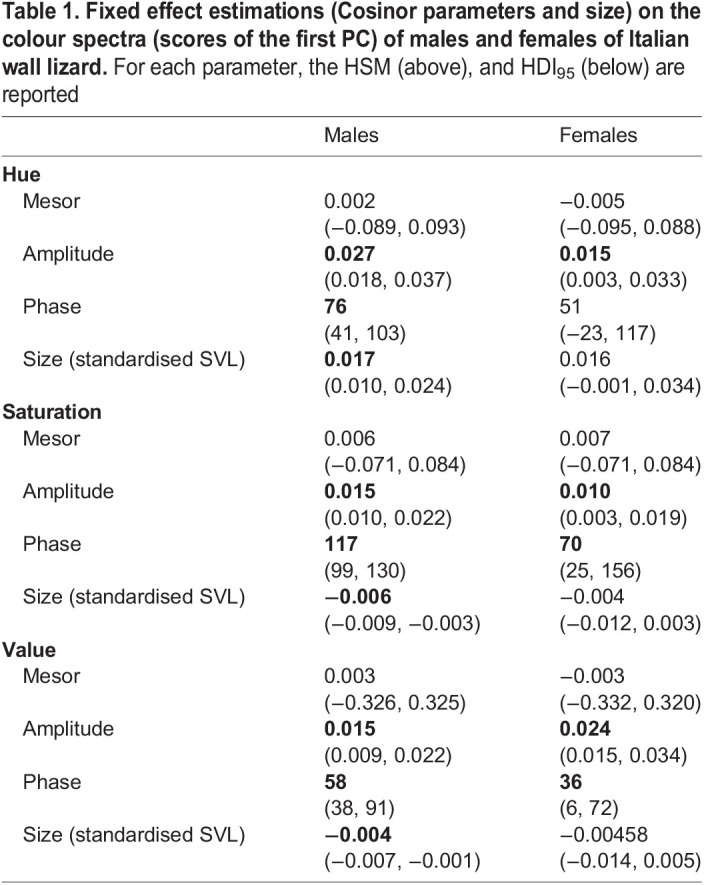
**Fixed effect estimations (Cosinor parameters and size) on the colour spectra (scores of the first PC) of males and females of Italian wall lizard.** For each parameter, the HSM (above), and HDI_95_ (below) are reported

The analysis of the Cosinor components for the PC1 of saturation confirmed that colour varied seasonally, as in this case also the amplitudes of both sexes deviated from zero (males: P_A>0_=0.99; females: P_A>0_=0.99, Table 1). Colour saturation increased during spring (Fig. 3B) and reached the maximum on 27 April in males (HDI_95_: 9 April, 10 May) and on 11 March in females (HDI_95_: 26 January, 6 June). As for hue, also the amplitude of saturation was higher in males than in females (Fig. 2B, *P*_m>f_=0.83). Concerning the effect of size, we observed male saturation to decrease with increasing size (Table 1; Fig. 4B), while no remarkable effect was detected for females (Table 1). Consequently, the posterior probability of having a larger effect of size in males than in females was 77% (Fig. 2C).

The last analysis on the PC1 score of brightness supplied further evidence for seasonal changes in colour expression (Fig. 3C). The amplitudes of the Cosinor deviated from zero in both males and females (males: *P*_A>0_=0.99; females: *P*_A>0_=0.99, Table 1), but with an opposite pattern with respect to hue and saturation. Indeed, the amplitude was larger in females than in males (*P*_m>f_=0.012, Fig. 2B). Males showed the maximum brightness on 28 February (HDI_95_: 7 February, 31 March), whereas females on 5 February (HDI_95_: 6 January, 12 March). As for hue and saturation, the effect of body size was observed only in males (Table 1): larger males were less bright than smaller ones (Fig. 4C). So, there was a marked difference between sexes regarding the effect of size on brightness (*P*_m>f_=0.95, Fig. 2C).

**Fig. 5.**
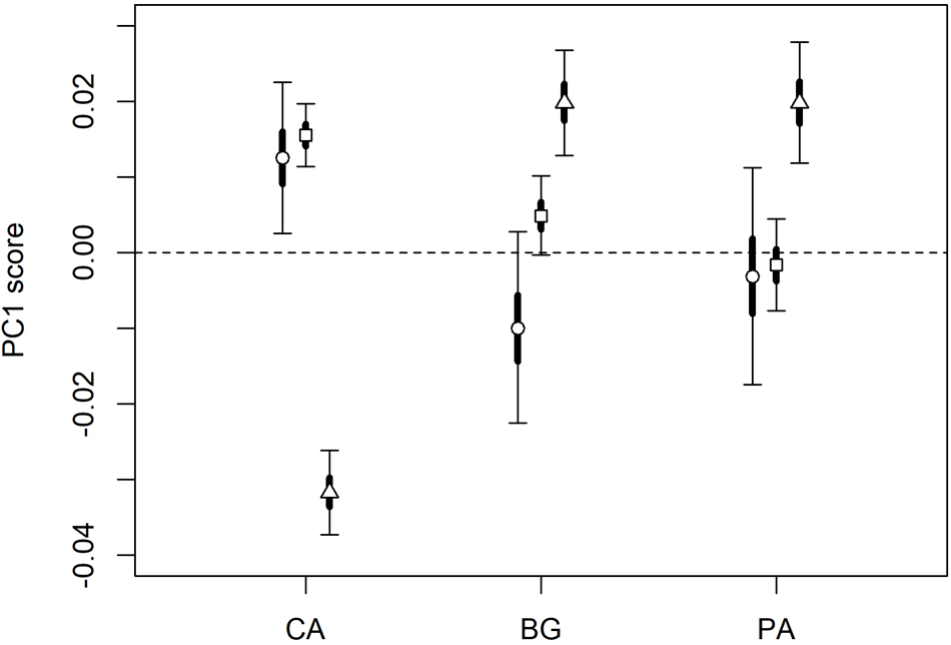
Posterior distribution values of the first component score of hue (circle), saturation (square) and brightness (triangle) for the three sampling sites (CA=Calci; BG=botanical garden; PA=Paola).

Finally, a large portion of the variance unexplained by sex, season, and body size depended on the population, especially for saturation and brightness. These proportions were 28.7% for hue (HDI_95_: 9.6%-82.6%), 42.1% for saturation (HDI_95_: 17.2%-91.3%), and 65.4% for brightness (HDI_95_: 36.1%-97.6%). Notably, lizards sampled in central Italy (i.e. Tuscany) were greener, more saturated, but less brilliant than those samples in southern Italy (i.e. Calabria) (Fig. 5).

## DISCUSSION

Our analysis demonstrates that the Italian wall lizard is characterised by a remarkable variability in terms of external body colouration, and that such variability is sexually and seasonally determined. Males were greener and more brilliant than females and showed a more pronounced seasonal pattern than females. In both males and females, the dorsal colouration appeared greener, more saturated and brighter during spring, whereas in autumn lizards of both sexes were browner, paler and duller. The role of ontogeny in the degree of green expression can be clearly detected only in males, which became greener, more saturated and less bright with increasing body size; while females did not show any clear pattern of colour expression concerning size.

Body colourations play a major role in the ecology of polymorphic species as they are deeply involved in a wide range of core functions and are strongly driven by selective pressures that can be summed up to the compromise between communication and crypsis (Galán, 2008; Cuthill et al., 2017). As a consequence, body colourations evolve according to the main function played and to the ecological constraints coped with. Therefore, conspicuous colourations will favour the maximization of intraspecific communication, notwithstanding the risk of being potentially detected by predators, while duller ones will be more likely to improve predator avoidance, thus relying on different ways to communicate with conspecifics (Cuthill et al., 2017). In agreement with past research on lizards that have outlined how conspicuous colourations have social significance for partner selection and male–male contests (LeBas and Marshall, 2000; Stuart-Fox et al., 2003; Husak et al., 2006; Bajer et al., 2010; Amdekar and Thaker, 2019), our data consistently indicate that males are more conspicuous than females for the whole duration of the reproductive season. This observation can be interpreted from different perspectives.

According to Pérez i de Lanuza et al. (2013) sexual size dimorphism (SSD) in lizards is largely driven by sexual selection as an outcome of male–male competition in sexually dimorphic lizards. Males of dimorphic species often show more complex, diverse and conspicuous colour patterns than females, which are instead duller and less visible; such sexual dichromatism also seems to be positively related to SSD. Hence it is reasonable to suggest that the males of *P. siculus* tend to be more conspicuously coloured with growing size as a potential signal of dominance towards competitors, thus favouring their potential mating success. Additionally, conspicuous colourations could also be involved in intersexual communication as male quality indicators (Cook et al., 2013; Pérez i de Lanuza et al., 2014). Coherently with these considerations, females of *P. siculus* are normally less colourful than males and their colour does not vary remarkably with age; this could be explained by the fact that an increase in colour expression, resulting in higher chances of being detected by predators, would reduce survivorship, and thus potential reproductive success (Bauwens and Thoen, 1981). From this perspective, it is known of other closely related European lacertids, like *Psammodromus algirus* and *Zootoca vivipara*, that male juveniles tend to show duller colourations than the adults, thus making them less detectable (Martin et al., 2013; Moreno-Rueda et al., 2021). Although it is relatively complex to determine the nature of such differences, it is reasonable to hypothesize that less conspicuous colourations are selected to favour concealment, similarly to what is displayed by females. Additionally, differential colour expression with dully coloured juveniles can also be addressed to the minimisation of predation risk, harassment by adults and to convey a signal of subordinance to adults (Lyon and Montgomerie, 1986; Hawkins et al., 2012).

When considering the effect of seasonality on colour expression patterns, it was possible to identify important patterns of periodic seasonal colour variations. Male lizards in particular showed more marked variations concerning all the three chromatic parameters of the HSV system, whereas such variations among females were mild (Table 2). The chromatic variation of males was almost in synchrony with the circannual period for H, S and V, thus indicating that colour expression (i.e. becoming greener and brighter) is tightly dependent on the phase of the activity period when specific colourations are necessary. Fig. 3A shows the effect of seasonality on colour variations for both sexes, where males tend to delay the maximum of green to the peak of the reproductive period (April–June) overlapping it with the minimum of saturation. As a consequence, males tend to be greener and brighter than females that, on the contrary, are darker throughout the whole activity period. According to these findings, it is reasonable to assume that in the Italian wall lizard two specific seasonal patterns can be identified: although both sexes show similar patterns of seasonal chromatic variations, on the one hand, males become globally more visible until the peak of reproduction, thus favouring the communicative aspect of colour expression, on the other hand females are generally more cryptic and less detectable (Marshall and Stevens, 2014; Marshall et al., 2015). This hypothesis is consistent with reported hormone seasonal variations of this species, where hormones reach their peaks simultaneously and overlap with the peak of green expression recorded in our research. Such hormones are in fact significantly involved in reproduction as both catecholamines and corticosterone (De Falco et al., 2004) and testosterone (Andò et al., 1990) are associated with aggressive behaviour that is most frequent during the reproductive period and especially in males. Therefore, it is possible that seasonal chromatic variations towards greener colourations are effectively associated with rising hormone levels in both sexes of *P. siculus.*

**Table 2.**
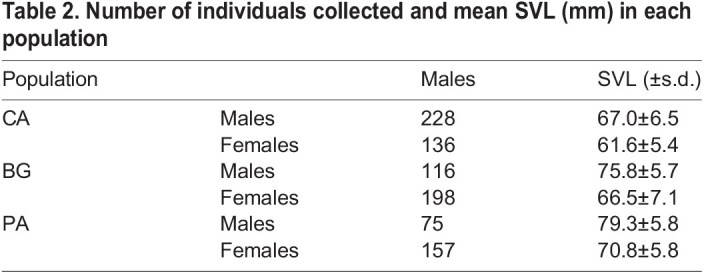
Number of individuals collected and mean SVL (mm) in each population

Furthermore, despite not being the focus of our research, the analyses highlighted the occurrence of variation among the studied populations. Notably, lizards from southern populations (Calabria) were brighter, but less green and saturated than those from central Italy (Tuscany). Although the cause of this discrepancy is unclear, we can hypothesize two potential reasons: it might be determined by a phylogenetic difference between two populations belonging to the separate clades *P. siculus campestris* (Tuscany) and *P. siculus siculus* (Calabria) (Senczuk et al., 2017) and resulting in morphological discrepancy in dorsal patterns (Corti et al., 2011); alternatively, darker patterns of Tuscanian lizards could be induced by environmental factors such as lower solar radiation and soil temperature (Pinna, 2017), which may affect colour expression in order to improve the efficiency of thermoregulation (Andrén and Nilson, 1981). In fact, in many colour shifting taxa (i.e., reptiles, amphibians, crustaceans and fish), temperature affects melanocyte-stimulating hormone determining melanin dispersion (Stuart-Fox and Moussalli, 2009). As reported by Norris (1967), reptiles can actively become darker or lighter and match background colouration according to environmental temperature, thus compensating lower thermal regimes with darker colourations to increase thermoregulatory efficiency. Hence, a similar adaptation can be addressed to the case of *P. siculus* northern populations that, under lower radiation and temperature conditions, might become darker to compensate for potentially less favourable environmental conditions.

In conclusion, our research points out that *P. siculus* is characterised by marked colour variations throughout the year, with distinct patterns between males and females, thus resulting in two diametrically different strategies, where the former is more communication-oriented and the latter is more crypsis-oriented. Since this is one of the first studies on the annual chromatic variations of *P. siculus*, further research is needed to extend our knowledge of the physiological and ecological mechanisms that could determine such variations (namely hormone secretion or pigment acquisition from the environment) and the extent to which they are perceived in the lizards’ visual spectrum. However, our experimental protocol is limited to assessing colour variations in the visible spectrum, thus indicating that this lizard matches the overall background of the environment in accordance with the findings of Pellitteri-Rosa et al. (2020). From this perspective, it is reasonable to assume that such differences in dorsal colourations are mainly camouflage-oriented to reduce detection by predators, given their ability to perceive the wavelengths within the visible spectrum. It would be important for future research to provide solid evidence supporting the assumption that lizards’ colour expression and their predators’ visual perception overlap, thus indicating that dorsal colourations could have a major role in camouflage. On the other hand, provided that lizards are known for being able to see ultra violet (UV) wavelengths as well and that ventral/lateral colourations are commonly used to communicate with conspecifics, the extant limited knowledge about UV signalling in the species we studied (Pérez i de Lanuza and Font, 2006; Stapley and Whiting, 2006) makes it still necessary to investigate in future research whether its body colourations also express UV signals and whether they play any role in intraspecific communication.

## MATERIALS AND METHODS

### Data sampling

We collected the field data in 2019 throughout the whole activity period of the Italian wall lizard, from March to October, in three populations from central and southern Italy; the first site was in Calci, Tuscany (CA, 43°43′N, 10°31′E), the second and third were both in the proximity of Cosenza (Calabria), respectively, the botanic garden of the University of Calabria (BG, 39°21′N, 16°13′E), and the town of Paola (PA, 39°21′N, 16°2′E). Sampling surveys were performed once a month in each site by five to eight researchers for three consecutive days each time. All lizards were captured by noosing or by hand and then moved to the laboratory for measurements and photographs. Firstly, individuals were sexed and measured for snout-to-vent length (SVL) and tail length (TL) using a ruler (1 mm accuracy). Tail injury or loss was also recorded along with the breaking point (if detectable). All juveniles (SVL <50 mm; Henle, 1988) were excluded from the data collection and released after capture.

According to published data and thanks to the effectiveness and strength of this approach in studying animal colourations (Stevens et al., 2007), we used digital photography to retrieve a thorough framework of dorsal colorations of the studied populations. The method is able to provide a fine distinction, which is highly repeatable and finer than the human eye (Villafuerte and Negro, 1998). Therefore, for each individual, we took high-resolution digital images of dorsal colouration using a Nikon D50 camera at a 1.2-million-pixel resolution, equipped with a Nikkor 60 mm AF-S Micro lens, and fixed on a stand at a distance of 18 cm. Each picture was taken adjacent to a GretagMacBeth Mini ColorChecker chart (24 colour references, 5.7×8.25 cm) in a 44×44 cm lightbox illuminated with two daylight 22 W circular neon tubes (Reporter 55100 Studio-kit). After data collection, all lizards were released in the spot where they were captured. Overall, we measured and photographed 910 distinct lizards, including 419 males and 491 females (Table 2).

### Colour analysis

We performed the analysis using the red-blue-green (RGB) colour system because colour vision is a common trait of diurnal vertebrates; although it has been extensively pointed out that the visual spectra of the different vertebrates can be highly variable due to the presence of an additional class of cones in birds (González-Martín-Moro et al., 2017) and reptiles (Fleishman et al., 1993), colour vision is present in lizards and in their predators, such as mammals or birds of prey (Kelber et al., 2003; Potier et al., 2018). Hence, this approach, despite not being reliable enough to assess the whole visual spectrum of both emitters (lizards) and receivers (conspecifics and predators) thoroughly, is adequate to provide partial insight about the potential constraints driving colour expression in our model species. We analysed the pictures according to the method by Bergmann and Beehner (2008) and Sacchi et al. (2013). Firstly, the Camera plug-in for Adobe Photoshop CS3 was used to generate a new colour profile that adjusted the colour in the photographs (jpeg format) to the known colour levels in each square of the ColorChecker chart. Then, for each image, we selected the region of interest (ROI) using the ‘lazoo’ tool, and pixels corresponding to point of reflected light were removed using the package ‘magick’ in R (Ooms, 2018). Subsequently, we analysed on average 59,806±44,296 pixels for each image, ranging from 2469 to 452,305 for the dorsal region. Eventually, the RGB colour values were rearranged in the hue, saturation, and value (HSV) system. It differs from the cubic geometry of the RGB colour space because it consists of a cylindrical-coordinate system in which the hue is the angle around the vertical axis and corresponds to the colour lights, the saturation is the distance from the axis, and the brightness corresponds to the distance along the axis.

### Statistical analysis

**Fig. 6.**
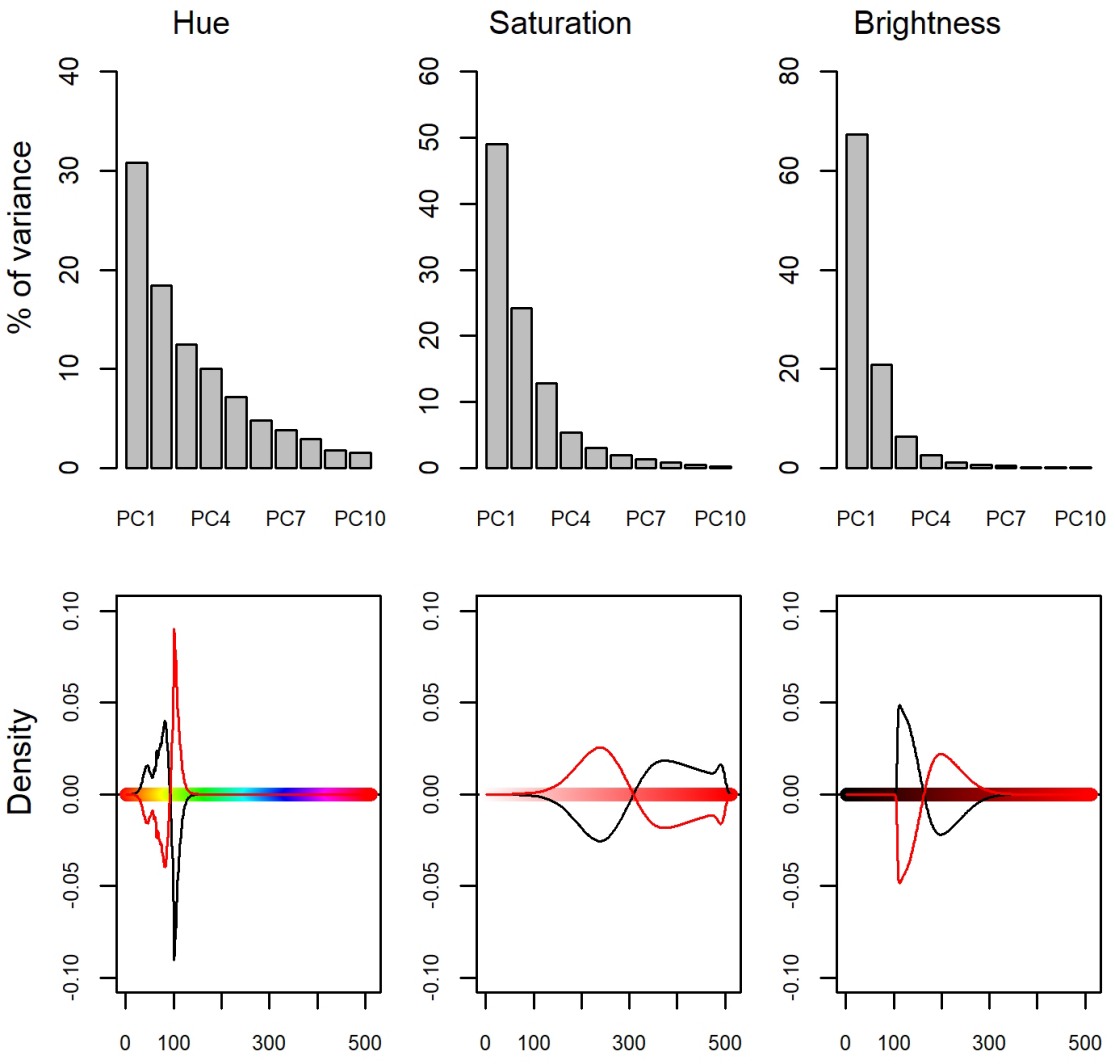
**Principal component (PC) analysis on hue, saturation and brightness of body colouration in the Italian wall lizards.** Upper panels report the amount of variance associated with the first ten PCs; lower panels show the effects of the PC1 from the lowest (black) to the highest (red) scores expressed as variation with respect the mean values (i.e. the mean spectrum reported in Fig. 1).

Colour variation in the Italian wall lizards was assessed in a three-step analysis (Sacchi et al., 2021). First, we generated the individuals’ frequency distributions of hue, saturation, and brightness by using values computed on the whole samples of pixels selected for each individual (HSV colour spectrum). Second, three principal component analyses (PCA), one each for H, S, and V, respectively, were used on the colour spectra, and the first components, explaining 30.7%, 49.0% and 67.2% of the total variance, were used as a proxy to summarise the inter-individual variability of colouration. The hue PC score accounted for the opposite variation of the orange-yellow and green interval in the hue colour spectrum; negative scores were associated with a higher peak in the orange-yellow interval whereas positive scores were associated with a higher peak in the green interval. The saturation PC score accounted for the increase of colour saturation with an increased score, whereas the value PC score accounted for the increase of brightness with higher scores (Fig. 6). Third, the PC scores were analysed through random intercept linear mixed models (LMM) including a single-component cosinor function to model the effect of the season (Refinetti et al., 2007; Cornelissen, 2014). Cosinor models were originally developed to model circadian rhythm in physiological processes (Halberg et al., 1967), but they can be also used to model periodic variations in ecological variables (Mangiacotti et al., 2019; Sacchi et al., 2020). In Cosinor models the response variable (Y) is assumed to depend on time (t) following a regular cycle, which is incorporated in a linear model through a cosine function:
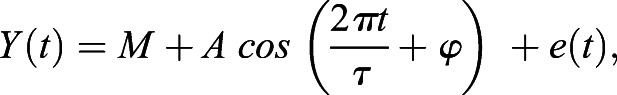
where M is the MESOR (Midline Statistic Of Rhythm, i.e. the time-corrected mean of the response), A is the amplitude (maximum absolute deviation from MESOR), τ the period of the cycle, 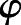 the acrophase (i.e. the timing of highest values), and e(t) the error term (Cornelissen, 2014). The model can be linearized by rewriting the formula: *Y*(*t*)=*M*+*βx*+*γz*+e(t); being *x*=*cos*(2*πt*/*τ*) and *z*=*sin* (2*πt*/*τ*) the cosinor terms, and *β*=*Acos*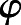 and *γ*=− *Asin*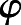 the cosinor coefficients (Cornelissen, 2014). In our model, the two cosinor terms entered the LMM as fixed effects, with time expressed as Julian date (1= 1 January) and τ=365 to account for circannual rhythms around the time-corrected mean of H, S, V values. Additional fixed effects were sex and body size (i.e. standardized SVL). The two-way interactions sex×size, sex×*x*, and sex×*z* were also added to account for possible differential effect of sex on size and season on colour expression. Population entered the model as a random effect. The dependent variables were the PC scores of H, S and V values, and three independent models were run. LMMs were fit in a Bayesian analytical framework available in the package JAGS 4.3.0 (http://mcmc-jags.sourceforge.net/), using flat priors for coefficients and intercept (μ=0 and σ=0.001), and uninformative half-Cauchy priors (x_0_=0, γ=25) for both σ²_error_ and σ²_population_. For all models, Markov Chain Monte Carlo parameters were set as follows: number of independent chains=three; number of iterations=34,000; burning=4000; thinning=three. Convergence was checked and results from the posterior distribution are reported as the half sample mode (HSM, Bickel and Frühwirth, 2006) plus 95% (or 50%) highest density intervals (HDI95; HDI50; Kruschke, 2010). In Bayesian statistics, the HSM is a commonly used estimator of the central tendency of posterior probability distribution robust to outliers, whereas the HDI_95_ defines the interval that includes the parameter with 95% probability. Parameter values in the centre of the HDI have higher credibility than parameter values at the limits of the interval. Therefore, when the HDIs of two groups do not overlap, there is a credible evidence for different group means. By contrast, to the extent of the two groups’ HDIs overlap there is no credible evidence of difference between the means. When comparing two groups (e.g. males and females), we therefore reported in Table 1 (bold) the posterior probability of their difference being different (i.e. higher or lower) from zero. Data preparation, model settings, call to JAGS, and posterior elaborations were done in R 4.0 using the package R2jags (Su and Yajima, 2015), modeest (Poncet, 2012), and HDInterval (Meredith and Kruschke, 2018).
